# A Practical Treatise on Dental Medicine

**Published:** 1852-10

**Authors:** 


*A Practical Treatise on Dental Medicine, being a compendium of Medical Science, as connected with the study of Dental Surgery: to which is appended an Inquiry into the use of Chloroform, and other Anæsthetic Agents. Second Edition, revised, corrected and enlarged.* By THOMAS E. BOND, A. M., M. D., Professor of Special Pathology and Therapeutics in the Baltimore College of Dental Surgery, pp. 366, 8vo. LINDSAY & BLAKISTON, Philadelphia, 1852.

SOON after the publication of the first edition of the above valuable book, we took occasion to express our opinion with regard to the merits of the work. It is exactly suited to the wants of the student and practitioner of dental surgery, and should be read by every physician. The information which it contains is as important to the one as to the other, and should be possessed by both. As a literary production it is equal to our very best works on general medicine.

The present edition is vastly superior to the first. Besides the additions interspersed throughout the entire work, a chapter on the use of anæsthetic agents is added, in which the author describes the circumstances under which they should be administered and the causes of the bad effects that have resulted from their employment.

